# Surgical Treatment of Secondary Pneumothorax-Complicated Interstitial Lung Disease

**DOI:** 10.7759/cureus.46816

**Published:** 2023-10-10

**Authors:** Ryusei Yoshino, Nana Yoshida, Nanami Ujiie, Akane Ito, Masaki Nakatsubo, Mishie Tanino, Masahiro Kitada

**Affiliations:** 1 Thoracic Surgery and Breast Surgery, Asahikawa Medical University Hospital, Asahikawa, JPN; 2 Diagnostic Pathology, Asahikawa Medical University Hospital, Asahikawa, JPN

**Keywords:** pneumothorax, steroid, surgery, secondary pneumothorax, interstitial lung disease

## Abstract

Introduction:To investigate the feasibility of early surgical treatment and perioperative steroid use in patients with interstitial lung disease (ILD) complicated by pneumothorax.

Methods: We retrospectively examined data, including patient characteristics, laboratory findings, surgical treatment details, postoperative complications, and deaths, of nine patients with ILD complicated by secondary pneumothorax. The patients had been treated at our hospital during the past 10 years.

Results: All nine patients were male (median age, 69.0 years). A total of nine patients had a histopathologic diagnosis of ILD after surgery. Of these, five were clinically diagnosed with ILD before surgery. Collagen disease was diagnosed in one case, drug-induced in one case, and idiopathic ILD (IILD) in three cases. All nine patients were diagnosed with postoperative ILD, including one case of collagen disease, one case of drug-induced, three cases of idiopathic pulmonary fibrosis (IPF)/cryptogenic fibrosing alveolitis, one case of nonspecific interstitial pneumonia (NSIP), and three cases of cryptogenic organizing pneumonia (COP). Regarding preoperative clinical characteristics, the performance status (PS) was 0 or 1 in all patients. Overall, three patients received oxygen (0-3 L/min), whereas steroids were administered to five patients. The mean drainage period was 23.5 days, and this was consistent with the time taken from pneumothorax occurrence to surgery. Video-assisted thoracic surgery (VATS) and thoracoscopic-assisted surgery were performed in seven and two patients, respectively. No postoperative recurrence or surgery-related deaths occurred.

Conclusions: Early surgery for secondary pneumothorax complicated by ILD may be a viable option for patients in good preoperative condition. For patients who are preoperatively treated with steroids, continued use of steroids should be carefully considered.

## Introduction

The term “interstitial lung disease (ILD)” is used to describe a group of diseases wherein inflammation and fibrosis (proliferation of fibrous tissue) occur in the interstitium of the lungs. In ILDs, the interstitium is damaged by chronic inflammation or abnormal tissue proliferation, and the normal structure and function of the lungs may be impaired. ILDs can be caused by known causes or associations such as drugs or occupational ILD, idiopathic ILDs (IILDs), connective tissue diseases associated with autoimmune diseases such as systemic lupus erythematosus, dermatomyositis, and polymyositis, or bacterial, viral, or fungal agents. The symptoms of ILD include dyspnea, which is more pronounced with exercise or exertion, chronic dry cough, fatigue, general malaise, and weight loss. The diagnosis of ILD is based on patients' medical history and physical examination findings. Tests of lung capacity, expiratory capacity, and oxygen exchange capacity are performed to assess lung function. Chest high-resolution computed tomography (HRCT) is also the most common imaging procedure and is useful in evaluating abnormal patterns and lesions in the lungs. A lung biopsy may be necessary to confirm the histopathologic diagnosis, which may include transbronchial lung biopsy or surgical lung biopsy. Treatment of ILDs is individualized based on the cause, progression, and severity of the condition; if ILDs are associated with a specific underlying disease (e.g., autoimmune disease, connective tissue disease), appropriate treatment of that underlying disease is indicated. Steroids and immunosuppressive agents (such as azathioprine and cyclosporine) may also be used to reduce immune system over-reactivity. Antifibrotic agents (such as pirfenidone and nintedanib) may be used for the treatment of ILDs with fibrosis, such as idiopathic pulmonary fibrosis (IPF), whereas oxygen therapy may be used in either setting [1-3].

Secondary pneumothorax may occur in patients with underlying diseases; it is the most common complication in patients with chronic obstructive pulmonary disease and the second most common complication in patients with ILD [4]. The incidence of pneumothorax in patients with ILD is reportedly 3.6-7.6% [5,6]. However, many patients have a low performance status (PS) before surgery. Moreover, patients who have been treated with steroids are susceptible to infection, with an associated risk of postoperative exacerbation of ILD. Therefore, the selection of surgical treatment is complicated in such cases [7,8], with generally poor surgical outcomes noted. To the best of our knowledge, no studies have reported on evaluating surgical intervention in the context of the preoperative condition and treatment with steroids in patients with pneumothorax secondary to ILD. The purpose of this study is to investigate the causal relationship between steroid treatment and surgery for pneumothorax secondary to ILD. This manuscript was prepared in accordance with the Strengthening the Reporting of Observational Studies in Epidemiology (STROBE) checklist.

## Materials and methods

This was a retrospective, observational study. Out of the 83 patients who underwent surgery for pneumothorax at Asahikawa Medical University Hospital, Asahikawa, Japan during the 10-year period from January 2012 to January 2022, nine patients (11%) with ILD (diffuse lung disease of any type) complicated by secondary pneumothorax were included in this study. Patients without preoperative pathology results diagnosing ILD were excluded. The clinical diagnosis of ILD was based on the 2002 American Thoracic Society (ATS)/European Respiratory Society (ERS) classification of IILDs and the 2011 official statement of the ATS/ERS/Japanese Respiratory Society/Latin American Thoracic Association on IPF [1]. ILDs are broadly classified into those caused by collagen disease/drugs; those caused by IILDs; those caused by granulomatous lesions, such as sarcoidosis; and those caused by other causes. IILDs are classified according to pathological features as IPF/cryptogenic fibrosing alveolitis, nonspecific ILD, cryptogenic organizing pneumonia (COP), acute ILD, desquamative ILD, respiratory bronchiolitis-associated ILD (RB-ILD), and lymphoid ILD [1]. Further, classification based on HRCT findings has included the four classifications in the Official ATS/ERS/JRS/ALAT Clinical Practice Guideline, 2018 [2]. This study examined patient characteristics, laboratory findings, surgical treatment details, postoperative complications, and death. PS was based on the definition of the Eastern Cooperative Oncology Group (ECOG), a US oncology organization. PS 0 indicates that an individual is completely safe and able to perform the same daily activities as before the onset of the disease, with no restrictions; PS 1, limited in physically strenuous activities but can walk and perform light or sedentary tasks; PS 2, ambulatory and able to perform all personal activities but is unable to work and spends more than 50% of the day outside the bed; PS 3, can only perform limited personal activities and spends more than 50% of the day in bed or in a chair; and PS 4, cannot move at all and is completely confined to a bed or chair. The study was conducted in accordance with the Declaration of Helsinki and approved by the Institutional Review Board of Asahikawa Medical University Hospital (protocol code 22113). The Institutional Review Board of Asahikawa Medical University Hospital waived the need for consent from individual patients owing to the retrospective nature of the study. Descriptive statistics were used as statistical methods. Continuous variables are expressed as mean and median values. No other statistical tests or techniques were used.

## Results

Table 1 summarizes the patient characteristics, surgical treatment details, and postoperative outcomes. This study included a total of nine participants; all of them were men, with a median age of 69 years. The median Brinkman Index was 1,350, and the median body mass index was 20.4 kg/m^2^. Five of the nine patients were noted to have ILD as an underlying disease, and one of them had collagen disease (polymyositis/dermatomyositis). Therefore, one patient was diagnosed with ILD due to collagen disease; three patients were diagnosed with IILDs (wherein all the three patients had a subtype of IPF); and one patient was diagnosed with ILD with a drug-induced cause. Of all patients, one patient had dementia as a comorbidity. Regarding preoperative clinical characteristics, the PS was 0-1 in all patients. Three of the nine patients required more than 1 L of oxygen preoperatively, and five patients received steroids. The mean drainage period was 23.8 days, and this was consistent with the number of days from the occurrence of the manual pneumothorax to the date of surgery.

**Table 1:** Clinical characteristics, surgical treatment details, and postoperative course of study patients. ave, average; med, median; BMI, body mass index; ILD, interstitial lung disease; IILDs, idiopathic interstitial lung disease; IPF/CFA, idiopathic pulmonary fibrosis/cryptogenic fibrosing alveolitis; NSIP, nonspecific interstitial pneumonia; COP, cryptogenic organizing pneumonia; AILD, acute interstitial lung disease; DILD, desquamative interstitial lung disease; RB-ILD, respiratory bronchiolitis-associated interstitial lung disease; LILD, lymphoid interstitial lung disease; PPFE, pleuroparenchymal fibroelastosis; PIE, pulmonary interstitial emphysema

Clinical characteristics of patients	
Cases	9
Male/female	9/0
Age (years)	69.3 (ave), 69.0 (med)
BMI (kg/m^2^)	21.4 (ave), 20.4 (med)
Brinkman Index	1,383 (ave), 1,350 (med)
Oxygenation	
Yes/no	3/6
ILD	
Yes/no	5/4
Collagen disease	1
Drug-induced	1
IILDs	3
Steroid use	
Yes/no	5/4
Dementia	
Yes/no	1/8
Preoperative drainage period (days)	23.8 (ave), 20.5 (med)
Surgical treatment	
General anesthesia	9
Operation	
Right/left	4/5
VATS/open	7/2
Covering	
Yes/no	7/2
Fibrin glue use	
Yes/no	7/2
Resection area	
Apex	7
Others	S4, S10/S8/apex, S3, S6
Operation time (minutes)	46 (ave), 43 (med)
Clinical diagnosis based on histopathology	
Collagen disease	1
Drug-induced	1
IILDs	
IPF/CFA	3
NSIP	1
COP	3
AILD	0
DILD	0
RB-ILD	0
LILD	0
Factors of pneumothorax inferred from pathological features	
Bullae	5
PPFE	1
PIE	3
Others	0
Postoperative course	
Duration of chest tube placement (days)	2.7 (ave)
Leak	
Yes/no	1/8
Postoperative complications	
Yes/no	0/9
Hospital stay (days)	9.4 (ave)
Mortality	0
Yes/no	0/9

Oxygen was administered to three patients, all of whom received <3 L/min of oxygen. Blood samples showed elevated Krebs von den Lungen-6 and surfactant protein D levels of 242-1,655 (mean, 885) U/mL and 97-258 (mean, 160) ng/mL, respectively. Liver and renal function parameters and electrolyte levels were within reference ranges. No significant increase was noted in the inflammatory response in any of the patients. Respiratory function tests were performed within three months before surgery in only three patients: one patient had a vital capacity (VC) of 1,650 mL; %VC, 50.9%; forced VC (FVC), 1,600 mL; %FVC, 51.4%; percent of diffusing capacity of the lungs for carbon monoxide (%DLco), 68.6%; and %DLco/Va, 81.1%. The second patient had a VC of 2,930 ml; %VC, 85.2%; FVC, 3,190 mL; %FVC, 99.7%; %DLco, 89.4%; and %DLco/Va, 79.6%. The third patient had a VC of 2,610 mL; %VC, 66.2%; FVC, 2,540 mL; %FVC, 72.6%; %DLco, 62.8%; and %DLco/Va, 72.9%. Preoperative HRCT findings showed usual ILD (UILD) in four patients (44%), probable UILD in two patients (22%), indeterminate UILD in two patients (22%), and alternative diagnosis in one patient (11%).

The surgery was performed under general anesthesia with one-lung ventilation in all patients. One-lung ventilation was established on the right side in four patients and on the left side in five patients. Video-assisted thoracic surgery (VATS) was performed in seven patients, whereas thoracoscopic-assisted surgery was performed in two patients. No patient required extracorporeal lung assistance. Partial pneumonectomy was performed for the lesions by bullectomy using an endo-stapler in all patients. The most common resection area was the apex in seven patients, and partial resection of two or more segments was performed in two patients (S4 and S10 in one patient; S1, S3, and S6 in the other patient). The wounds were covered with a polyglycolic acid (PGA) sheet and fibrin glue in seven patients (78%). The mean operative time was 47 (25-69) minutes.

The mean postoperative drainage period was 2.7 (2-7) days, and the mean length of postoperative hospital stay was 8.4 (6-18) days. A postoperative air leak was observed in one patient. In this patient, pleurodesis was performed with autologous blood transfusion, tetracycline, and OK-432 on postoperative day 3, and the drain was removed on postoperative day 5. No postoperative recurrence was observed in any patient, and no surgery-related deaths occurred.

Preoperative pathology results in all nine patients resulted in a diagnosis of ILD. The condition was determined to be collagen-related in one of these patients and drug-related in another patient. The other seven patients (78%) were diagnosed with IILDs, with three patients (33%) having IPF/cryptogenic fibrosing alveolitis, one patient (11%) having nonspecific interstitial pneumonia (NSIP), and three patients (33%) having COP by subtype. Pathology results further showed that bullae caused pneumothorax in five patients (56%), pleuroparenchymal fibroelastosis (PPFE) in one patient (11%), and pulmonary interstitial emphysema (PIE) in three patients (33%).

Five patients (56%) were on steroid therapy prior to surgery: one was on prednisolone (PSL) 30 mg/day, which was tapered weekly to 20 mg/day, 15 mg/day, and 12.5 mg/day by the day of surgery; and another was on PSL 10 mg/day and the immunosuppressive agent azathioprine (100 mg/day). One patient was taking 10 mg/day of PSL and 100 mg/day of azathioprine, an immunosuppressant drug. One patient was on PSL 30 mg/day, which was tapered over the course of a week to 25 mg/day, 20 mg/day, and eventually 15 mg/day by the day of surgery. One patient was on PSL 55 mg/day, which was tapered weekly to 40 mg/day, 30 mg/day, and 20 mg/day by the day of surgery.

All five patients received intravenous steroids on the day of surgery, equivalent to the amount of PSL administered one day prior to surgery. Postoperatively, the PSL was resumed at the same dose as the PSL administered one day prior to surgery and was tapered upward in 5-10 mg increments on a monthly basis.

## Discussion

IILDs include a group of ILDs of unknown cause. There are several major subtypes of IILDs; IPF is the most common subtype of IILDs and is characterized by progressive pulmonary fibrosis. Generally, it is more common in middle-aged smokers older than 50 years. HRCT shows a peripheral, subpleural, and basal distribution; reticular, honeycombing traction bronchiectasis/bronchiolectasis; architectural distortion; and focal ground-glass features. Pathologic features show a UILD pattern. NSIP is mainly characterized by interstitial inflammation and fibrosis and shows a different pattern from IPF. The symptoms of NSIP are similar to those of IPF, but progression is relatively slow and the prognosis is better than that of IPF. HRCT shows a peripheral, subpleural, basal, and symmetric distribution, with features of ground-glass attenuation and irregular lines. Lung consolidation characterizes COP, which is an inflammatory lung disease involving the formation of fibrous emboli in the alveoli. HRCT shows a subpleural/peribronchial distribution and features patchy lung consolidation and/or nodules. Pathologic features include an organized pneumonia pattern. Acute ILD is a rapid and severe ILD that can cause respiratory failure in a short period of time. The cause is unknown, but infection and environmental factors have been implicated. HRCT shows a diffuse distribution, lung consolidation, and ground-glass opacity, often with lobular sparing. RB-ILD is a subtype of IILDs associated with smokers and mainly affects young adults with a history of smoking, presenting with symptoms such as sputum, dyspnea, and dry cough. It is characterized by bronchial wall thickening, centrilobular nodules, and patchy ground-glass opacity. Pathological features include a respiratory bronchiolitis pattern. These subtypes of IILDs are distinguished based on histopathologic and clinical features. Accurate diagnosis and appropriate treatment are based on the specific IILD subtype involved; therefore, evaluation by several specialized physicians is crucial [1].

ILD may cause secondary pneumothorax through the destruction and fibrosis of the alveolar structure. In general, surgical treatment is challenging in patients with ILD complicated by pneumothorax. Moreover, patients with ILD are refractory to treatment because of reduced pulmonary compliance due to pulmonary fibrosis and a prolonged drainage period due to failure to re-expand the lungs. Furthermore, postoperative recurrence is as high as 30-80% [9,10]. Patients with ILD who are administered steroids are susceptible to infection. In addition, patients on steroids are at an increased risk of postoperative exacerbations of ILDs [7,8]. Many patients have low presurgical PS; Kawai et al. [11] reported preoperative PS as an independent risk factor for postoperative complications in patients undergoing surgery for secondary pneumothorax. Considering the high risk of postoperative complications in patients with a preoperative PS of 3 or higher, surgical intervention should be carefully considered.

These findings suggest that early surgical treatment can be considered for ILD complicated by secondary pneumothorax in patients with favorable preoperative clinical characteristics.

Moreover, Nishimoto et al. [12] reported risk factors in patients with pneumothorax and connective tissue disease-associated ILDs (CTD-ILDs). ILD is a common complication in CTD, such as rheumatoid arthritis, polymyositis/dermatomyositis, and Sjögren's syndrome. In these patients, the occurrence of pneumothorax is correlated with a poor prognosis, a low body mass index, and a high degree of reticular abnormalities determined by HRCT. Methylprednisolone pulse therapy is associated with the development of pneumothorax. In the present study, patients with pneumothorax due to collagen disease-related causes displayed a low body mass index and extensive reticular abnormalities observed via HRCT. Further, other patients showed consistent findings, with extensive reticular abnormalities diagnosed via HRCT. These findings are consistent with the results of previous reports of secondary pneumothorax in patients with IPF [13]. Therefore, the presence of extensive reticular abnormalities observed via HRCT suggests a higher risk of pneumothorax development. Whether this applies to IILDs in general is an area for further study. Pneumothorax and mediastinal emphysema have been implicated in the development of PIE, a condition in which emphysema occurs within the interstitium of the lungs. While emphysema usually occurs within the alveoli, in PIE, air leaks are noted into the interstitium of the lung tissue, resulting in abnormal air diffusion [14]. Tachibana et al. [15] found that PIE was a poor prognostic factor in ILD patients and a risk factor for air leaks in pneumothorax and mediastinal emphysema. In this study, postoperative histopathology revealed that the most common cause of pneumothorax was lung tissue due to bullae, followed by PIE and PPFE. Although limited by the small number of cases, this study advocates the need to search for pathological causes of pneumothorax in the future.

In the perioperative period, if possible, tapering of steroid doses should be considered. Patients with ILD are associated with an increased risk of lung cancer, and lung resection for lung cancer patients with ILD may cause acute exacerbation of ILD [13]. Some reports have also cited steroid use as an independent risk factor [13]. Ito et al. [16] performed a prophylactic study of acute exacerbations of ILDs after lung cancer surgery and found that the perioperative use of low-dose methylprednisolone was safe but did not prove effective in preventing the disease. However, there is currently no solid consensus among the many studies on the relationship between prophylactic steroid use and acute exacerbations of ILD after lung resection. Eastridge et al. [17] reported that steroid therapy delayed wound healing at the surgical site and weakened the pleura and cyst walls. This may occur because steroids inhibit tissue repair in lung and pleural injuries in all patients. Moreover, the probability of progression to refractory thoracic empyema with bronchopleural fistula is higher with than without steroid use because of increased susceptibility to infection. In such cases, plombage with a pedicle omental flap or latissimus dorsi muscle flap must be considered [18]. Thus, steroid therapy is believed to correlate with a high pneumothorax recurrence rate. Meanwhile, surgery for pneumothorax in patients with ILD has shown good results, and it is desirable to consider the indications for surgery carefully [19]. In this study, the group of patients who were being treated with steroids had their steroid dosage tapered to a minimal level by the date of surgery. Patients should be definitely considered for preoperative steroid reduction whenever possible; however, some patient conditions make reduction in steroid dosage challenging. This is because of the aforementioned risk factors for acute exacerbation of ILDs, with previous studies [17] showing that steroid use delays wound healing and increases the rate of recurrence. Although the small sample size and the lack of other studies have hampered a consensus on the method of tapering steroid dosage, we believe that our study may serve as useful reference in this regard. In this study, wounds were covered with a PGA sheet and fibrin glue in seven (78%) patients. We believe that these materials were useful for shortening the operative time and allowing complete closure of the pulmonary fistula [20,21].

This study has some limitations. First, the sample size was small, and the influence of chance errors could not be ruled out. In addition, the amount of steroids used was not the same across patients, and the regimen of dosage tapering was not standardized. Second, the short follow-up period of most recent surgical cases may have led to the underestimation of adverse event occurrence. Furthermore, there was a lack of objective factors used to decide the number of patients who underwent early or delayed surgery. Therefore, the external validity of the results may be limited.

Nevertheless, to the best of our knowledge, this is the first study to demonstrate the perioperative tapering of the steroid dosage prior to surgery for ILD complicated by secondary pneumothorax. Patients with ILD complicated by secondary pneumothorax may have a longer time until surgery owing to concerns about complications.

Based on the results of this study, early surgery for ILD complicated by secondary pneumothorax should be considered if the preoperative PS of patients is favorable. However, steroid use should be carefully considered. We expect that future studies focused on surgery for ILD complicated by secondary pneumothorax will be undertaken to shorten the time from admission to surgery and to manage steroid use.

## Conclusions

Considering the options for surgical treatment when devising a strategy for managing secondary pneumothorax complicated by ILD, early surgery can be a possible choice for patients with favorable clinical characteristics before surgery. Although patients treated with steroids are susceptible to infection, tapering the steroid dosage during the preoperative period may be useful, depending on the condition of each individual patient.
